# PARTICIPATORY RESEARCH APPROACH FOR EVALUATING HOME ENVIRONMENT FOR OLDER ADULTS WITH DISABILITIES

**DOI:** 10.1093/geroni/igac059.1999

**Published:** 2022-12-20

**Authors:** Juliana Tissot, Widya Ramadhani, Lizandra Vergara, Wendy Rogers

**Affiliations:** Federal University of Santa Catarina, Florianópolis, Santa Catarina, Brazil; University of Illinois Urbana-Champaign, Urbana, Illinois, United States; Federal University of Santa Catarina, Florianópolis, Santa Catarina, Brazil; University of Illinois Urbana Champaign, Champaign, Illinois, United States

## Abstract

Regardless of where they live, older adults’ ability to retain their independence depends on their health and capacity. Considering that older adults are more likely to experience disabilities, there are opportunities for built environmental intervention to help them conduct everyday activities to achieve successful aging in place. We conducted participatory research with designers, architects, and aging specialists to identify aspects and characteristics of the physical space that can support or limit older adults’ ability to do everyday activities. The research was conducted at the McKechnie Family LIFE Home, a home simulation facility. Participants were asked to walk through the home spaces and identify potential challenges and solutions for older adults with different disabilities (i.e., mobility, cognitive, sensory, socio-emotional), followed by a group discussion. Preliminary findings showed that the bathroom and kitchen had the most barriers. Recommendations to improve bathroom safety include installing high friction flooring, grab bars on both toilet sides, adequate wheelchair maneuvering space, and color contrast. Cooking was mentioned as a difficult activity and solutions included appliances at waist level, layout in “C” or a “U”, lights under cabinets, and in the foot space. Also, the cabinets doors should be translucent to allow them to check what is inside. The house floor plan should be designed with considerations of users’ everyday activities and the relationship between these activities, such as having the bathroom nearby bedroom, laundry nearby the kitchen to improve users ‘mobility. We have developed a protocol for designers to follow when evaluating home spaces.

